# Nomogram Model to Predict the Probability of Ovarian Hyperstimulation Syndrome in the Treatment of Patients With Polycystic Ovary Syndrome

**DOI:** 10.3389/fendo.2021.619059

**Published:** 2021-08-06

**Authors:** Fei Li, Ying Chen, Aiqin Niu, Yajing He, Ying Yan

**Affiliations:** ^1^Center for Reproductive Medicine, The First People’s Hospital of Shangqiu, Henan, China; ^2^Center for Reproductive Medicine, The First Affiliated Hospital of Zheng Zhou University, Henan, China; ^3^Department of Pathology, The First People’s Hospital of Shangqiu, Henan, China; ^4^Department of Molecular Biology, The First People’s Hospital of Shangqiu, Henan, China

**Keywords:** assisted reproductive technology, polycystic ovary syndrome, ovarian hyperstimulation syndrome, risk factors, norman model

## Abstract

**Objective:**

The objective of this study was to explore the risk factors of ovarian hyperstimulation syndrome (OHSS) in patients with polycystic ovary syndrome (PCOS) undergoing *in vitro* fertilization/intracytoplasmic sperm injection (IVF/ICSI) and to establish a nomogram model evaluate the probability of OHSS in PCOS patients.

**Methods:**

We retrospectively analyzed clinical data from 4,351 patients with PCOS receiving IVF/ICSI in our reproductive medical center. The clinical cases were randomly divided into a modeling group (3,231 cases) and a verification group (1,120 cases) according to a ratio of about 3:1. The independent risk factors correlation with the occurrence of OHSS was identified by logistic regression analysis. Based on the selected independent risk factors and correlated regression coefficients, we established a nomogram model to predict the probability of OHSS in PCOS patients, and the predictive accuracy of the model was measured using the area under the receiver operating curve (AUC).

**Results:**

Univariate and multivariate logistic regression analyses showed that FSH (OR, 0.901; 95% CI, 0.847–0.958; P<0.001), AMH (OR, 1.259; 95% CI, 1.206–1.315; P<0.001), E2 value on the day of hCG injection (OR, 1.122; 95% CI, 1.021–1.253; P<0.001), total dosage of Gn used (OR, 1.010; 95% CI, 1.002–1.016; P=0.041), and follicle number on the day of hCG injection (OR, 0.134; 95% CI, 1.020–1.261; P=0.020) are the independent risk factors for OHSS in PCOS patients. The AUC of the modeling group is 0.827 (95% CI, 0.795–0.859), and the AUC of the verification group is 0.757 (95% CI, 0.733–0.782).

**Conclusion:**

The newly established nomogram model has proven to be a novel tool that can effectively, easily, and intuitively predict the probability of OHSS in the patients with PCOS, by which the clinician can set up a better clinical management strategies for conducting a precise personal therapy.

## Introduction

For patients with polycystic ovary syndrome (PCOS), polyfollicular development during controlled ovarian hyperstimulation (COH) in IVF/ICSI occurs easily, which is a difficult problem, and this aroused a general concern in assisted pregnancy by human-assisted reproductive technology (ART) (1). Ovarian hyperstimulation syndrome (OHSS) is a common iatrogenic complication occurring after ovulation hyperstimulation, which seriously affects the physical and mental health of patients and increases the complications in the perinatal period of pregnant women (2). A study showed that in the PCOS group, 22.1% had ovarian hyper stimulation syndrome (OHSS). Also, only 4.7% had OHSS in non-PCOS group (p<0.05) (3). Owing to the threshold window of follicle stimulating hormone (FSH), which is difficult to control, the risk of OHSS is greatly increased in patients with PCOS infertility receiving COH in IVF/ICSI (4). Therefore, early detection and prevention in advance of OHSS in PCOS patients are extremely important to the safety of COH treatment.

There are many studies regarding the high-risk factors of OHSS in patients with PCOS, those high-risk factors may include the following: age, body mass index (BMI), race, body height, anti-Mullerian hormone (AMH), antral follicle count (AFC), and so on. However, a study showed that the AMH level does not reduce the incidence of OHSS for women with PCOS undergoing an ART treatment (5). Another study showed that patients with a higher value of BMI, free androgen, AFC, follicle number on the day of hCG injection and AMH are more likely to develop OHSS, but not confined to PCOS patients (6). Several studies showed that obese patients with PCOS have a higher OHSS probability than normal-weight patients, and overweight and high serum total cholesterol were risk factors for the outcome of IVF/ICSI cycles in PCOS patients (7). However, the predictive value and clinical usefulness of these variable factors are still controversial (8, 9). Considering the physical hazards and psychological burden imposed by OHSS on infertile patients, exploring accurate and reliable independent risk factors, which affect the occurrence of OHSS, is necessary.

A nomogram is a tool that provides graphical depictions of all variables in the model and enables the user to easily compute output probabilities. Up to now, there is no report indicating logistic regression-based nomogram model that has been used to predict the probability of OHSS in patients with PCOS. Therefore, identifying different risk factors by logistic regression analysis (using patients derived clinical characteristics and laboratory parameters) to establish a special nomogram model may be an essential option for predicting the probability of OHSS. By using the predictive model, clinicians can not only predict the probability of OHSS but also formulate controlled ovarian hyperstimulation and personalized treatments, which might be leading to a better clinical outcome and improved safety in the PCOS patients receiving the IVF/ICSI treatment.

At our reproductive medical centers, > 10000 IVF/ICSI cycles are performed each year, which can help us more conveniently to screen out independent predictive factors for the occurrence of OHSS and construct the nomogram model according to the high-risk factors. This logistic regression-based nomogram model results in the quantified, visualized, and graphical-based calculation of those variable risk factor values, combined with continuously displayed prediction probability, which would lead to the prediction of OHSS probability more accurately, and it can be used for early prevention and treatment of polycystic ovary syndrome.

## Materials and Methods

There were 4,351 PCOS patients receiving IVF/ICSI treatment included in this retrospective study. The patients were treated at the reproductive medical center of the First Affiliated Hospital of Zhengzhou University and the Shangqiu First People’s Hospital from March 1, 2014, to October 30, 2018. Each PCOS patient was identified by their unique medical record number, and these clinical cases were randomly divided into a modeling group (3,231 cases) and a verification group (1,120 cases) according to a ratio of about 3:1. The random grouping principle used an automated random number generator in SPSS 22.0. The study project has been reviewed and approved by the hospital IRB committees. Diagnosis and treatment strategy designing was conducted by experienced senior physicians. The patient data were collected and reviewed by specialists and double checked by two additional investigators to ensure accuracy and complete for quality control.

Patients selection and inclusion criteria were according to the Rotterdam criteria, at least possessing two of the following three criterions can be defined as PCOS: (1) rare ovulation or chronic anovulation; (2) having hyperandrogenemia or hiperandrogen clinical manifestation; (3) demonstrating polycystic ovarian changes by ultrasound determination and exclusion of other etiologies (4, 6). Patients with the following exclusion criteria were included: IVF/ICSI contraindications, uterine malformations, chromosomal abnormalities, rheumatic immune system diseases, untreated endometrial lesions, drug allergies, severe mental system diseases, pituitary tumors, congenital diseases, and abnormalities of blood glucose, blood pressure, thyroid function, prolactin, and so on.

The patients were put on either the GnRH-agonist protocol or GnRH antagonist protocol for IVF/ICSI followed by embryo transfer in the same cycle. The initial dose of gonadotropin (Puregon, Organon, The Netherlands) was formulated based on the patient’s sinus follicle count, age, BMI, and response to the previous ovarian stimulation cycle. Then, the doses of gonadotropin could be sequencely adjusted according to the patient’s responses. According to the guidelines of the Chinese Medical Association, when dominant follicles measuring > 16 mm in diameter accounted for 60% of all follicles, or when a follicle reached 20 mm in mean diameter and three follicles > 17 mm, 250 µg of human chorionic gonadotropin (Livzon Pharmaceuticals, China) was used to trigger injection. 37 hours after injection, eggs were collected under vaginal ultrasound guidance. After embryo transfer, corpus luteum support therapy was given, progesterone soft capsules (French apricot pharmacy) 0.2 g, vaginal administration, twice a day. The number of embryos transferred varied from one to two based on the principle of the Health Ministry of China.

For the purpose of our study, we have used the following criteria to define ovarian hyper-stimulation syndrome (OHSS), as long as any of the following diagnoses are met, we assume that the patient has OHSS: (a) ovary enlargement, bloating, mild abdominal pain, ovary diameter <8 cm; (b) severe bloating, nausea and vomiting, presence of ascites, and the ovarian diameter is 8 to 12 cm; (c) ascites (or pleural effusion), oliguria (< 300 ml/d or < 30 ml/h), HCT > 0.45, hyponatremia (sodium < 135 mmol/L), low osmolality (< 282 mmol/L), hyperkalemia (potassium >5 mmol/L), hypoproteinemia (serum albumin < 35 g/L), ovary diameter > 12 cm; (d) tension ascites/large pleural effusion, HCT > 0.55, WBC > 15 × 10^9^/L, oliguria/anuria, vascular embolism, acute respiratory distress syndrome (7–9).

### Statistical Analysis

All analyses were performed using the statistical packages R (The R Foundation; http://www. R-project.org; version 3.6.1) and SPSS 22.0 (IBM, Armonk, NY, USA). The continuous variable data are expressed as the mean ± standard deviation and were compared using Student’s t test or the Wilcoxon rank sum test, categorical variables are expressed as percentages and were compared using the chi-square test. The relevant factors affecting the final live birth rate were determined by logistic regression analysis and P<0.05 was considered to be a statistically significant effect, which is considered as an independent influencing factor that affects OHSS. Based on the results of multi-factor logistic regression analysis and the regression coefficients of related variables, the corresponding nomogram was constructed to be a graphic representation of the prediction model with the R language software.

The predictors included in the multivariable model were pre-selected based on knowledge from the existing literature, in this study, variables entered into the model were the FSH, AMH, E2 values on the day of hCG injection, total dosage of Gn used, and follicle number on the day of hCG injection. The points for each variable are summed on the total points line. Then, a vertical line is projected from the total points line, which corresponds to the individual probability obtained from OHSS.

The prediction performance of the nomogram model was verified by using Bootstrap equivalent repeated sampling 1000 times. In addition, the receiver operating characteristic (ROC) curve was used to determine the best critical value, corresponding sensitivity and specificity. The area under the ROC curves was used to qualify the accuracy of test results: 0.50 to 0.70 of the area under ROC was set to as a low accuracy, 0.71 to 0.90 is medium accuracy, and >0.90 is high accuracy. P<0.05 indicates that the difference is statistically significant.

## Results

Based on the clinical characteristics and laboratory analysis, 986 patients were classified into the OHSS group, and 3,365 patients were in the non-OHSS group. The results of comparison of the baseline clinical and laboratory data are listed in **Table 1**. In comparison of 15 parameters between the OHSS group and non-OHSS group, eight have statistically significant difference, which include FSH, AMH, AFC, total dosage of Gn used, duration of Gn used, E2 value on the day of hCG injection, follicle number on the day of hCG injection, and oocyte number (**Table 1**).

**Table 1:** Comparison of clinical characteristics in the PCOS patients with and without OHSS.

Factors	OHSS group (n=986)	Non-OHSS group (n=3365)	*t/x^2^* value	*P* value
Age (years)			1.095 0.579	
<30	62.7 (618/986)	64.3 (2164/3365)		
30-35	32.2 (317/986)	30.4 (1023/3365)		
>35	5.1 (51/986)	5.3 (178/3365)		
Infertility years			0.991 0.609	
<3	29.1 (287/986)	29.6 (996/3365)
3-4	39.1 (386/986)	37.4 (1260/3365)
>4	31.8 (313/986)	33 (1109/3365)
BMI (Kg/M2)	24.26 ± 3.462	24.12 ± 4.181	1.020	0.308
FSH (IU/L)	5.640 ± 1.472	5.8577 ± 1.647	−3.706	<0.001
Basal LH (IU/L)	9.771 ± 6.663	9.355 ± 6.899	1.661	0.097
Basal E2 (ng/L)	69.75 ± 288.605	74.80 ± 362.898	−0.398	0.691
Basal P (μg/L)	0.699 ± 1.438	0.767 ± 1.789	−1.073	0.283
AMH (ng/mL)	8.48 ± 4.080	5.42 ± 2.497	28.808	<0.001
AFC(n)	22.41 ± 4.589	21.93 ± 4.982	2.699	0.007
No. of treatment cycles			0.145 0.703	
≤1	52.4 (517/986)	51.7 (1739/3365)
>1	47.6 (469/986)	48.3 (1626/3365)
Total dosage of Gn used	2101.79 ± 970.693	1997.24 ± 893.265	3.168	<0.001
Duration of Gn used	13.97 ± 3.190	13.50 ± 2.926	4.315	<0.001
E2 value on the day of hCG injection	6355.3 ± 2538.789	6014.2 ± 1545.565	5.179	<0.001
follicle number on the day of hCG injection	24.05 ± 9.693	18.56 ± 5.377	22.961	<0.001
Oocyte number	22.29 ± 11.256	16.56 ± 6.500	20.197	<0.001

Data are shown as means ± standard deviation. BMI, body mass index; FSH, follicular-stimulating hormone; LH, luteinizing hormone; E2, estradiol; P, progesterone; AMH, anti-Müllerian hormone; AFC, antral follicle counting; Gn, Gonadotropin.

Multiple univariate logistic regression analysis demonstrated that FSH (OR, 0.901; 95% CI, 0.847–0.958; P<0.001), AMH (OR, 1.259; 95% CI 1.206–1.315; P<0.001), E2 value on the day of hCG injection (OR, 1.122; 95% CI, 1.021–1.253; P<0.001), total dosage of Gn used (OR, 1.010; 95% CI, 1.002–1.016; P=0.041), and follicle number on the day of hCG injection (OR, 0.134; 95% CI, 1.020–1.261; P=0.020) are the independent risk factors for OHSS development (**Table 2**).

**Table 2:** Logistic regression analysis of factors related to OHSS.

Factors	Unadjusted			Adjusted		
	OR	95% CI	*P* value	OR	95% CI	*P* value
FSH (IU/L)	0.916	0.875–0.960	<0.001	0.901	0.847–0.958	0.001
AMH (ng/mL)	1.361	1.323–1.399	<0.001	1.259	1.206–1.315	<0.001
AFC(n)	1.022	1.006–1.038	0.007	1.002	0.983–1.022	0.804
Total dosage of Gn used	1.032	1.013–1.051	<0.001	1.010	1.002–1.016	0.041
Duration of Gn used	1.053	1.028–1.078	<0.001	1.029	0.977–1.084	0.282
E2 value on the day of hCG injection	1.116	1.102–1.131	<0.001	1.122	1.021–1.253	<0.001
follicle number on the day of hCG injection	1.113	1.101–1.125	<0.001	1.134	1.020–1.261	0.020
Oocyte number	1.088	1.078–1.098	<0.001	0.966	0.882–1.058	0.460

FSH, follicular-stimulating hormone; AMH, anti-Müllerian hormone; AFC, antral follicle counting; Gn, gonadotropin.

**Figure 1:**
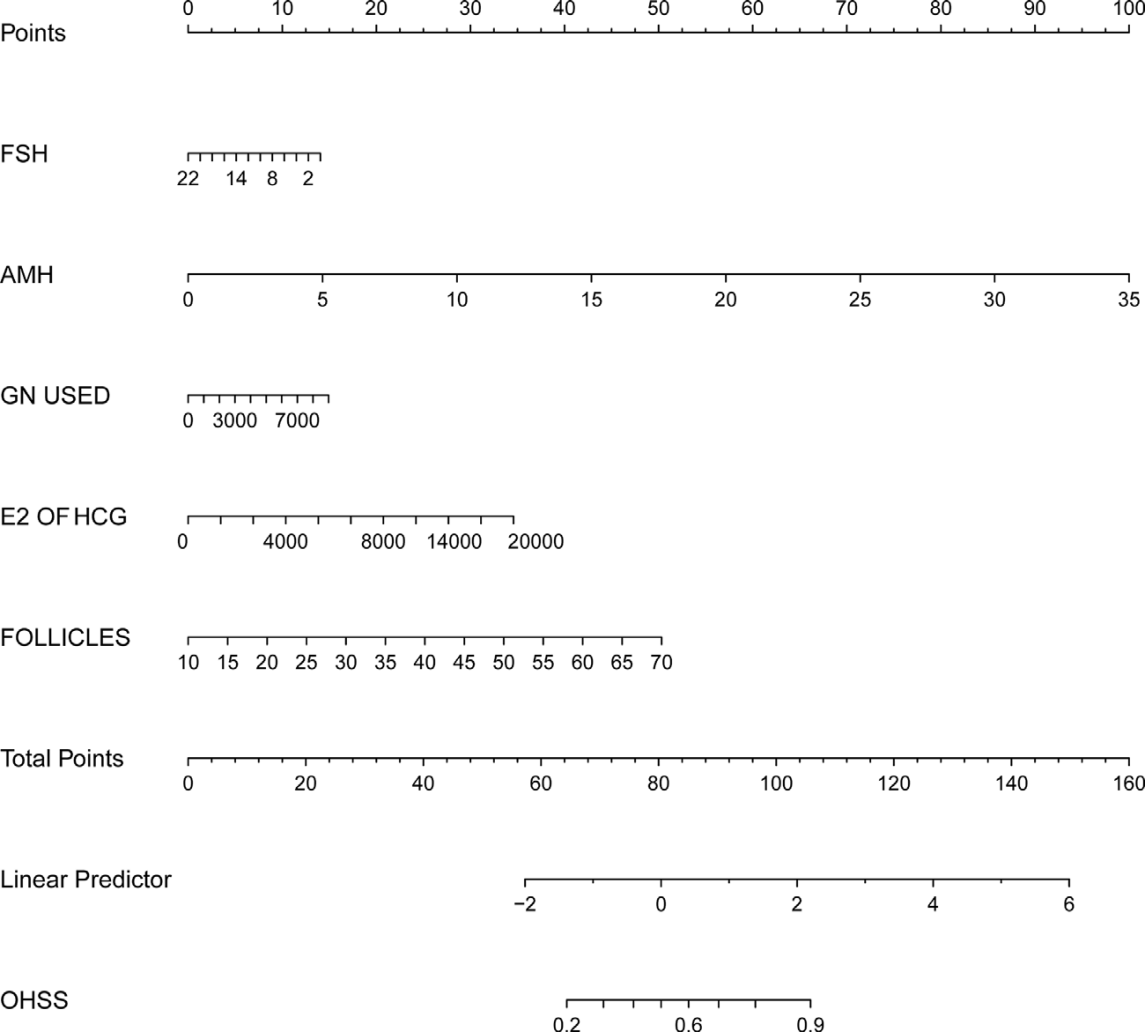
Nomogram to predict the probability of OHSS in PCOS related infertility patients. The probability of OHSS is calculated by drawing a line to the point on the axis for each of the following variables: FSH, AMH, E2 value on the day of hCG injection, total dosage of Gn used and follicle number on the day of hCG injection. The points for each variable are summed and located on the total points line. Next, a vertical line is projected from the total points line to the predicted probability bottom scale to obtain the individual probability of OHSS.

On the basis of the univariate and multivariate logistic regression analyses we have shown above, a nomogram prediction model incorporating the significant risk factors was established to predict the involvement probability of OHSS **(** **Figure 1** **)**.

The equation describing the probability of OHSS was as follows: *P*= −3.47315 to 0.05521 × FSH+0.24700 × AMH+0.00014 × total dosage of Gn used +0.00015 × E2 value on the day of hCG injection +0.07249 × follicle number on the day of hCG injection.

Using the nomogram prediction model, a table was composed to obtain the individual scores of each independent predictor. A regression model was established to generate a regression coefficient for each variable, and the generated maximum absolute value of coefficient was converted at 100 score. All other variables were divided by this maximum, then multiplied by 100 to convert into the corresponding score. These scores were cumulatively summed, and the total summed score will be used to predict the probability of each influencing factor.

**Figure 2:**
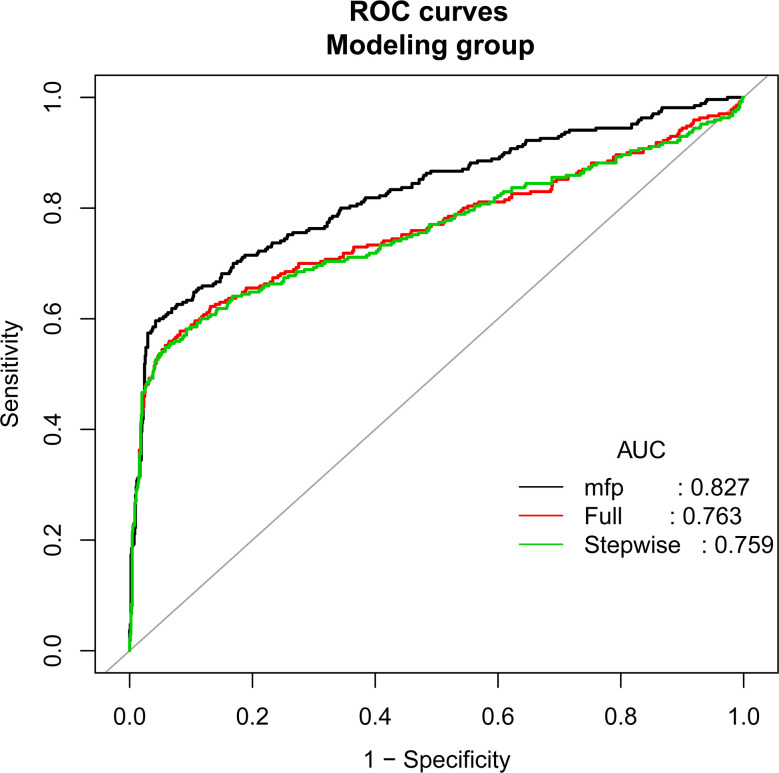
Discrimination for the training cohort. The AUC (mfp) of the modeling group is 0.827 (95% CI=0.795~0.859).

**Figure 3:**
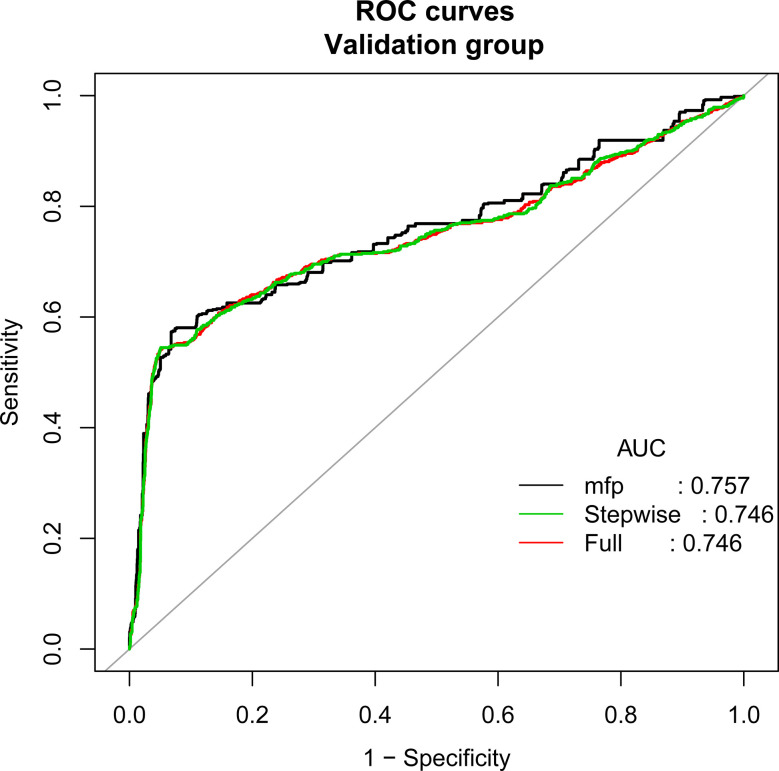
Discrimination for the training cohort. The AUC (mfp) of the verification group is 0.757 (95% CI=0.733~0.782).

According to the principle of 3:1 random sampling, patients were divided into a modeling group (3231 cases) and a verification group (1120 cases). There was no statistically significant difference in baseline data between the two groups (**Table 3**). Prediction accuracy of OHSS development in the patients with PCOS was evaluated by the calculation of AUC. The results demonstrated that the AUC was 0.827 (95% CI, 0.795–0.859), the specificity was 0.9578, the sensitivity was 0.7963, and the accuracy was 0.8777, respectively, indicating that the prediction model has a good conformity. In the calibration curves of the modeling group with verification group, the standard curve and the calibration prediction curve fit well, indicating that the predicted value and the observed value derived from this model are in good compliance (**Figures 2**, **3**).

**Table 3:** Baseline characteristics of patients in the modeling and validation groups.

Factors	Modeling group (n = 3231)	Validation group (n = 1120)	T value	*P* value
Age (years)			0.157 0.925	
<30	63.1 (2039/3231)	63.8 (714/1120)		
30-35	31.7 (1024/3231)	31.2 (348/1120)		
>35	5.2 (168/3231)	5.0 (58/1120)		
Infertility years			0.085 0.959	
<3	29.3 (947/3231)	29.6 (332/1120)
3-4	38.2 (1234/3231)	38.3 (429/1120)
>4	32.5 (1050/3231)	32.1 (359/1120)
BMI (Kg/M2)	24.18 ± 4.158	24.06 ± 3.635	0.824	0.410
FSH (IU/L)	5.90 ± 1.645	5.73 ± 1.475	0.488	0.626
Basal LH (IU/L)	9.26 ± 7.059	8.86 ± 6.044	0.698	0.506
Basal E2 (ng/L)	78.30 ± 393.01	71.43 ± 148.27	1.456	0.176
Basal P (μg/L)	0.80 ± 1.709	0.88 ± 1.728	-1.951	0.147
AMH (ng/mL)	6.10 ± 3.204	6.16 ± 3.204	-0.487	0.626
AFC(n)	21.91 ± 5.154	22.39 ± 4.055	-1.825	0.199
No. of treatment cycles			0.010 0.921	
≤ 1	52.1 (1683/3231)	52.3 (586/1120)
>1	47.9 (1548/3231)	47.7 (534/1120)
Total dosage of Gn used	2139.43 ± 895.442	2256.07 ± 920.135	-1.125	0.267
Duration of Gn used	13.92 ± 2.981	14.50 ± 2.849	-1.822	0.207
E2 value on the day of hCG injection	6076.39 ± 1.848	6135.19 ± 1.752	-0.930	0.353
follicle number on the day of hCG injection	19.75 ± 6.985	19.95 ± 7.023	-0.815	0.415
Oocyte number	17.80 ± 8.204	18.03 ± 8.161	-0.797	0.426

BMI, body mass index; FSH, follicular-stimulating hormone; LH, luteinizing hormone; E2, estradiol; P, progesterone; AMH, anti-Müllerian hormone; AFC, antral follicle counting; Gn, Gonadotropin.

## Discussion

PCOS is one of the most common endocrine diseases of women in the childbearing age, and it is also a major risk factor for OHSS development in artificial reproductive therapy (ART). Preventing the occurrence of OHSS in the patients with PCOS has always been a tough and hotspot in the clinical research of the ART (4, 10). The response to follicle-stimulating hormone during controlled ovulation in the patients with PCOS may be fluctuated and may be variable. Beginning with a low dose, follicle-stimulating hormone administration may result in ovarian unresponsiveness or only a single follicle development, however, a slightly higher dose of follicle-stimulating hormone may lead to an excessive ovarian recruitment, which makes the patients face a high risk of OHSS (11). Although successful controlled ovarian hyperstimulation without OHSS occurring is an urgent problem to be solved, a practical prediction model for the ART clinical treatment is still absent so far. In this study, we have established a nomogram model to predict the risk of OHSS development in the PCOS patients during controlled ovarian hyperstimulation treatment. By integrating relevant influencing factors, we transformed the logistic regression data into quantifiable, visualization, and graphical outcomes. The graphics were used to calculate the value of an individual variable and predict the probability of the eventual occurrence of OHSS in PCOS patients. The nomogram model established by us demonstrates a favorable prediction accuracy and specificity with an AUC of 0.827 (95% CI, 0.795–0.859). Compared with other prediction models of ART, the nomogram model is more intuitive and practical, and more convenient for clinicians to calculate the probability of OHSS risk according to the patient’s personal situation. In addition, it is possible to provide a more substantial judgment for the formulation of clinical interventions.

The pathogenesis of OHSS development is not clear so far. Patients with OHSS may manifest as blood volume reduction and blood concentration, hypovolemic shock, hypoperfusion of blood flow, which may cause liver and kidney function failure in severe cases (12, 13). In our study, an objective and accurate prediction nomogram model for OHSS was set up and validated in infertility patients with PCOS. Multiple univariate logistic regression analysis demonstrated that FSH, AMH, E2 value on the day of hCG injection, total dosage of Gn used and follicle number on the day of hCG injection are independent risk factors for OHSS development, these results are similar to previous studies. A study showed that patients with a higher value of natural follicle count, preovulatory follicles, and AMH are more likely to develop OHSS (8). Another study showed that the basal serum AMH level predicted OHSS better than age, and BMI with a specificity of 81.3%. Both the basal serum AMH level (odds ratio, 1.7856; P = 0.0003) and serum E2 level on the day of hCG injection administration (odds ratio, 1.0005; P = 0.0455) proved to be significant predictors of OHSS by logistic regression analysis (9). Furthermore, some studies have demonstrated that the high risk factors associated with OHSS mainly include (6, 14): (1) low age (age <35 years); (2) low body weight; (3) polycystic ovary syndrome; (4) overdoses of exogenous ovarian stimulation during ovulation induction; (5) high plasma levels of estrogen; (6) having a history of OHSS (15, 16). Studies have also shown that multiple follicles developed at the same time during the ovulation induction cycle, higher number of eggs obtained in the ART cycle and higher doses of exogenous human chorionic gonadotropin administration during the ovulation induction cycle, as well as pregnancy, may lead to an increased risk of OHSS (17). Follicles numbers on the day of hCG injection are believed to be an excellent indicator for predicting the occurrence of OHSS; however, this predictive factor seems to have a relatively low specificity (0.69) (18).

In a report in 2014, Steward et al. analyzed 256,381 ovulation induction cycles, found that the number of eggs retrieved >15 could significantly increase the risk of OHSS (19). Kwee et al. found that AFC is another major risk factor for predicting OHSS. When AFC>14, the sensitivity to predict the occurrence of OHSS is 0.82, and the specificity is 0.89 (20). In another study, Ng et al. found that when AFC>9, the sensitivity of OHSS risk is 60%, and the specificity is 71% (21). An RCT study by Lee et al. in 2007 showed that AMH and hCG injection doses are better predictors for OHSS than age and BMI. When AMH concentration >3.62 g/L and E2 value on the day of hCG injection >1431 ng/L, the two parameters are significant predictors on the development of moderate to severe OHSS (22). In addition, Tarlatzi et al. found that when the E2 value on the day of hCG injection is over 8077.67 pmol/L, the possible sensitivity of severe OHSS occurring is 85.0% and specificity is 71.8% (23). Studies by Griesinger et al. also showed that E2 level on hCG daily administration is an effective indicator for prediction of severe OHSS in the patients with PCOS. The sensitivity and specificity are 62.3% and 63.6%, respectively (24). The study of Ashrafi et al. found that when hCG daily dose E2 >7505.2 pmol/L, predictive sensitivity of moderate/severe OHSS is 96.5% with a specificity 83.7% (25). In our study, five independent risk factors were found to be associated with the development of OHSS, this is roughly consistent with the conclusions of some previous studies. Because these risk factors that correlated to the occurrence of OHSS have been successfully screened, the nomogram model may provide a much more precise and accurate prediction for the patients with OHSS.

According to our study results, over dose of exogenous ovulation-stimulating drugs is one of the high-risk factors for OHSS development. Therefore, the minimum dose of gonadotropin achieving therapeutic effect should be a consideration principle of the ovulation-stimulation strategy. In addition, ultrasound detection of follicular development and serum estradiol levels are important parameters in dynamic monitoring of OHSS occurring, it is necessary to monitor the ovarian reserve and endocrine status from the initial clinical basic state, then periodically examine the follicular development and serum estradiol level during the ovulation induction cycle, to avoid or reduce the probability of OHSS (26, 27). Furthermore, overmuch of follicle numbers is a high-risk factor for the OHSS development, similar to the study of Jayaprakasan et al, in which their result suggests that the numbers of follicle, which is 24, is significantly correlated with the incidence of moderate to severe OHSS (28). Based on some previous studies, formulating an appropriate controlled ovarian hyperstimulation for each individual patient should be tailored according to the patient’s personal situation and not advisable to use more eggs as a successful indicator in an ovulation procedure. In addition, for the patients with high risk of OHSS, appropriate use of whole embryo freezing can reduce the production of endogenous hCG, thereby decreasing the production of vasoactive substances and effectively avoiding the occurrence of severe OHSS (29–31).

The main limitation of our study is its retrospective nature, which cannot exclude all potential biases. Although in our attempts to remove confounding factors and screen eligible subjects according to the SOP criteria, but with selection bias being introduced, one might expect the result to be skewed in some ways. It is worth emphasizing that past studies have shown that Rotterdam criteria, in the context of IVF/ICSI, do not necessarily reflect the actual women at high risk of OHSS, as this has been shown to be more reliably predicted by AFC or AMH alone. Therefore, there was a high proportion of women that have not had OHSS despite being labeled as POCS, and in this study, our research subjects are Chinese PCOS patients with infertility, and we exclude incomplete laboratory data or other ovarian hyperstimulation protocols, therefore, the findings of this study cannot be used for everyone, there is a certain deficiency in the universality and extrapolation of research. Despite the limitations, results in this study suggest that our OHSS predicting nomogram, which warrants further investigation for us. Nomogram could be a useful tool in helping physicians, as well as the PCOS patients, to decide on a treatment option before IVF/ICSI.

In summary, the nomogram model has been proven to be a novel tool that can effectively, easily, and intuitively predict the probability of OHSS in the patients with PCOS, which can offer optimal clinical management strategies to the clinicians for individual therapy. Compared with the traditional logistic regression model, this nomogram is simple, intuitive, practicable, and valuable in clinical applications, which however, warrants further observations.

## Data Availability Statement

The original contributions presented in the study are included in the article/supplementary material. Further inquiries can be directed to the corresponding author.

## Ethics Statement

The studies involving human participants were reviewed and approved by the ethics committee of Shangqiu First People’s Hospital. Written informed consent for participation was not required for this study in accordance with the national legislation and the institutional requirements.

## Author Contributions

FL designed, conducted and supervised the general study. FL and YC responsed for the patients selection and classification. AN and YH reviewed and classified laboratory data for the patients. FL and YY reviewed and FL drafted and composed the manuscript. All authors contributed to the article and approved the submitted version.

## Funding

This work was supported by the National Natural Science Foundation of China (81771534), the Key Science and Technology Foundation of Henan Province (212102310049) and the Medical Science and Technology Co-construction Project of Henan Province(LHGJ20200933).

## Conflict of Interest

The authors declare that the research was conducted in the absence of any commercial or financial relationships that could be construed as a potential conflict of interest.

## Publisher’s Note

All claims expressed in this article are solely those of the authors and do not necessarily represent those of their affiliated organizations, or those of the publisher, the editors and the reviewers. Any product that may be evaluated in this article, or claim that may be made by its manufacturer, is not guaranteed or endorsed by the publisher.
